# Gastroenteritis Caused by Norovirus GGII.4, the Netherlands, 1994–2005

**DOI:** 10.3201/eid1301.060800

**Published:** 2007-01

**Authors:** J. Joukje Siebenga, Harry Vennema, Erwin Duizer, Marion P.G. Koopmans

**Affiliations:** * National Institute for Public Health and the Environment, Bilthoven, the Netherlands; † Erasmus University, Rotterdam, the Netherlands

**Keywords:** Norovirus, molecular epidemiology, caliciviruses, gastroenteritis, outbreak, dispatch

## Abstract

From 1994 through 2005, gastroenteritis outbreaks caused by norovirus generally increased in the Netherlands, with 3 epidemic seasons associated with new GGII.4 strains. Increased percentages of GGII.4 strains during these epidemics, followed by a sharp decrease in their absolute and relative numbers, suggest development of immunity.

Noroviruses (NoVs) cause large outbreaks of gastroenteritis in settings of close human contact such as hospitals, institutions, military bases, and cruise ships, as well as sporadic cases. In recent years, human NoVs have increasingly been recognized as a common cause of gastroenteritis. Since the introduction of rapid molecular detection techniques, a high proportion of acute gastroenteritis outbreaks have been attributed to NoVs (*1**–**3*). We describe trends in occurrence of NoV in the Netherlands, with a focus on the predominant GGII.4 strains.

## The Study

Surveillance of viral gastroenteritis outbreaks was initiated just over a decade ago at the National Institute for Public Health and the Environment in the Netherlands (RIVM). Outbreaks were typically reported by the municipal health service or the food inspection services to the RIVM, and samples were collected in close collaboration with these agencies. To determine the role of NoV and possible differences between different NoV strains in gastroenteritis outbreaks, a minimal set of epidemiologic data (setting, date of onset, number of persons affected, most probable mode of transmission, and number of hospitalizations) was collected for reported outbreaks. These data were supplemented with results of molecular biologic detection and typing techniques to enable more in-depth analysis of surveillance data.

Preliminary typing of strains was performed by sequencing region A (280 nt in the polymerase gene) of the virus. A systematic selection of strains was also typed by sequencing region C (277 nt in the capsid gene) (*1**,**2*). Because NoV activity is much higher in winter months, seasons were analyzed from July through June, rather than per calendar year.

From December 18, 1993 to December 26, 2005, a total of 1,032 gastroenteritis outbreaks were reported to the RIVM. Samples from 942 outbreaks were received and analyzed. Of these, 695 (74%) outbreaks met our inclusion criteria for a NoV outbreak (≥25% of samples positive by reverse transcription–PCR). Overall, we observed an increasing trend in the number of reported outbreaks per year (Figure, panel A). In the 1995–96, 2001–02, 2002–03, and 2004–05 seasons, more NoV outbreaks were reported (66, 90, 154, and 161, respectively). GGII virus strains were predominant in all years and caused 577 (91%) of 631 outbreaks with known genotypes, compared with 36 (6%) of 631 outbreaks caused by GGI or GGIV viruses and 18 (3%) of 631 outbreaks caused by mixed infections with viruses of different genotypes (Table).

**Table:** Outbreaks of gastroenteritis caused by noroviruses per mode of transmission and setting, the Netherlands, 1994–2005*

Genotype	Mode of transmission per genotype, no. (%)					Setting per genotype, no. (%)							
	MW	F	PTP	U/O	Total	T	S/D	R/C/C	H	PH	RI	U/O	Total
GGI + IV	1 (50)	5 (11)	7 (3)	23 (5)	36	3 (25)	3 (9)	9 (16)	4 (3)	2 (67)	10 (2)	5 (11)	36
GGII.4	0	18 (39)	163 (73)	291 (69)	472	7 (58)	11 (34)	20 (36)	91 (74)	1 (33)	316 (74)	26 (58)	472
GGIInon4	0	14 (30)	33 (15)	58 (14)	105	2 (2)	10 (31)	16 (29)	17 (14)	0	55 (13)	5 (11)	105
Mixed	1 (50)	3 (7)	4 (2)	10 (3)	18	0	4 (13)	2 (4)	3 (2)	0	6 (1)	3 (7)	18
Unknown	0	6 (13)	17 (8)	41 (10)	64	0	4 (13)	8 (15)	8 (7)	0	38 (9)	6 (13)	64
Total	2 (100)	46 (100)	224 (100)	423 (100)	695	12 (100)	32 (100)	55 (100)	123 (100)	3 (100)	425 (100)	45 (100)	695

*MW, mainly waterborne; F, foodborne; PTP, person-to-person; U/O, unknown or other; T, travel associated; S/D, school or daycare center; R/C/C, restaurant, canteen, or caterer; H, hospital; PH, private house; RI, residential institution.

GGII.4 strains have been detected since 1995, with the highest proportions observed in years with high numbers of outbreaks. In the epidemic seasons of 1995–96, 2002–03, and 2004–05 the percentages of outbreaks caused by GGII.4 were 82%, 83%, and 89%, respectively, compared with an overall average of 68% (Figure). In seasons after these epidemics, the percentage caused by GGII.4 decreased to 39% in 1996–97, 55% in 2003–04, and 32% in the first half of 2005–06. Multiple NoV genotypes co-circulated throughout the years of the study, but in postepidemic years, outbreaks caused by non-GGII.4 strains were more common (Figure, panel C). The high number of outbreaks in 2001–02 may be partially explained by emergence of a new variant of GGII.4 in the spring of 2002 (*4*), which caused uncharacteristically high numbers of outbreaks between April and June. The epidemic increases in the number of outbreaks and seasonality of outbreaks were mainly attributable to GGII.4. Strains with genotypes other than GGII.4 were found at similar levels throughout the year (data not shown).

**Figure:**
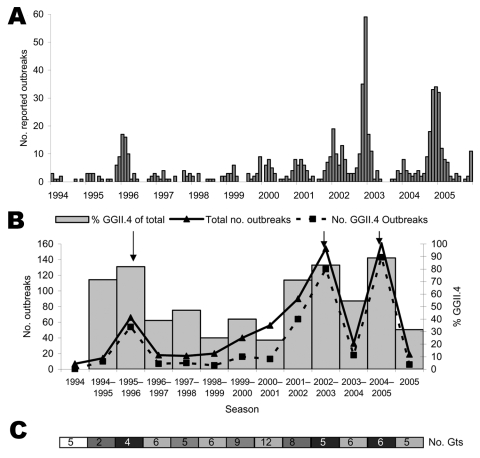
A) Number of norovirus outbreaks reported per month in the Netherlands, 1994–2005. B) Total no. of outbreaks per season and fraction of GGII.4 outbreaks reported in the Netherlands. Total no. is indicated by the solid line, no. of GGII.4 outbreaks by the dotted line (values on left y-axis), bars indicate percentage of GGII.4 outbreaks of the total no. (values on right y-axis), and arrows indicate epidemic seasons. Seasons run from July through June. C) Total no. of genotypes (Gts) circulating per season. Shading of the bar indicates the percentage of GGII.4, ranging from white (0%–20%), in steps of 20%, to black (80%–100%).

A total of 548 (79%) outbreaks were reported in healthcare settings (hospitals and residential institutions) compared with 102 (15%) in nonhealthcare settings; for 45 (6%) outbreaks no data were available (Table). A total of 407 (81%) of 502 outbreaks with genotyping information in healthcare settings were caused by GGII.4 NoV strains (Table). In the nonhealthcare settings, GGII.4 was significantly less prevalent (39 [43%] of 91, p<0.0001), resulting in a relative risk at least 2.17× higher for acquiring a GGII.4 infection in a healthcare setting than in other settings.

A mode of transmission was reported for 272 (39%) outbreaks. Data confirmed that the main transmission route was person to person (60%, 163/272) (Table) (*5**,**6*). GGII.4 strains were found in 73% of person-to-person outbreaks compared with 44% of food-related outbreaks (p<0.0001). When outbreaks for which no genotype was known were counted as non-GGII.4 strains, the relative risk of finding GGII.4 in an outbreak caused by person-to-person transmission was 2.3× greater than finding it in a foodborne outbreak or relative to other genotypes. Multiple GGII NoV strains were found in 18 outbreaks (3 foodborne, 1 waterborne, 4 person-to-person, and 10 with unknown modes of transmission).

## Conclusions

Detailed molecular epidemiologic data from long-term surveillance on NoV outbreaks are rare because NoV molecular detection techniques became available only in the mid-1990s. In our 12-year surveillance study, we observed large differences in the magnitude of the annual winter peak of NoV infection. All epidemic peaks were associated with predominance of GGII.4 strains. Although this finding has been observed in studies covering a shorter period (*3**,**5**–**7*), our data suggest an increase in infections with GGII.4 in recent years, particularly in healthcare settings. However, the number of outbreaks reported in healthcare settings is likely overrepresented in our study because of mandatory reporting of illness in such settings. An actual increase in the number of outbreaks cannot be proven based on passive surveillance data alone, but an increase is strongly suggested by increased prevalence of GGII.4 in recent years and supported by reported shifts in the predominant GGII.4 variant associated with large numbers of outbreaks (*8*).

The overall dominance of GGII.4 suggests that this genotype is more transmissible than other genotypes in healthcare settings, where close contact of many persons favors person-to-person transmission. Transmission may also be affected by poorer hygiene or greater susceptibility to infection. Increased transmissibility could result from increased levels of shedding of GGII.4 or altered stability of virus particles outside the host compared with other genotypes. Alternatively, changes in circulating viruses may lead to differences in host cell binding or immune recognition, thereby changing the dynamics of infection or size of the population at risk.

Our group and other researchers have reported the emergence of distinct GGII.4 lineages in 1995–96, 2002, and 2004 (*2*,*4*,*9*,*10*). This suggests that the changing phenotype of GGII.4 strains results in increased numbers of outbreaks (*10*). A detailed characterization of GGII.4 strains is ongoing to determine the molecular mechanisms involved in observed epidemiologic patterns. The marked decrease in the percentage of GGII.4 strains during seasons after epidemic seasons caused by variant strains suggests that populations may acquire immunity against these predominant strains.

The value of this surveillance dataset will increase with its continuation, as well as with its expansion as part of a European surveillance network (http://www.eufoodborneviruses.co.uk/). Future research will be directed at understanding the molecular basis for observed changes in the epidemiology of NoV and control of its spread.
